# Factors that influence the availability of childhood vaccine in healthcare facilities at Tana River County, Kenya

**DOI:** 10.1186/s40545-023-00648-8

**Published:** 2023-11-13

**Authors:** Billy Said Mkamba, Eugene Rutungwa, Peter Ndirangu Karimi, Joseph Lune Ngenzi

**Affiliations:** 1 EAC Regional Centre of Excellence for Vaccines, Immunization, and Health Supply Chain Management, College of Medicines and Health Sciences, University of Rwanda, Kigali, Rwanda; 2 Faculty of Health Sciences, University of Nairobi, Nairobi, Kenya; 3 School of Business, College of Business and Economics, University of Rwanda, Kigali, Rwanda; 4 School of Medicine and Pharmacy, College of Medicine and Health Sciences, University of Rwanda, Kigali, Rwanda

**Keywords:** Factors, Availability, Childhood vaccine, Healthcare facilities

## Abstract

**Background:**

Routine vaccine is a cost-effective health intervention against vaccine preventable diseases (VPD). Tremendous gains have been realized since the introduction of vaccines. Despite the gains, access to the lifesaving commodity has remained a major obstacle globally. Various factors have been associated with vaccine stock-out. This research assessed the factors that influence the availability of vaccines in healthcare facilities at Tana River County in Kenya.

**Methods:**

Cross-sectional design was adopted. Census sampling technique was used where all 61 immunizing healthcare facilities were included. The study was carried out in Tana River County which is located in the coastal part of Kenya. A structured questionnaire was used to collect the data. The researchers requested for authorization from relevant bodies and consent from participants. Data were collected, cleaned and recorded in Microsoft excel. STATA version 14 was used to analyze data. Both descriptive and inferential statistics were used in the analysis at 0.05 level of significance.

**Results:**

The study revealed that 62.71% of the facilities experienced routine vaccine stock-out. There was statistically significant association between availability of vaccines and work experience (*p* = 0.001), training on immunization services (*p* = 0.027), catchment area map with target population displayed in the facility (*p* = 0.049), and use of target population method in vaccine forecasting (*p* = 0.004). The independent predictor of vaccine availability was work experience (*p* = 0.025).

**Conclusion:**

There was inadequate vaccine forecasting, vaccine stock management practices and accountability. Work experience was the main factor that affected their availability in the health facilities.

## Background

Vaccination is a highly cost-effective measure against vaccine preventable diseases (VPD). It has contributed significantly to global health improvements [1]. Vaccines have played a pivotal role in eradicating and controlling life-threatening diseases worldwide [2, 3]. Between 2000 and 2018, measles vaccination prevented 23.2 million deaths globally [4]. A total of 37 million deaths due to VPD were averted between 2000 and 2019 worldwide [5]. However, challenges persist in ensuring universal access to routine vaccines. Between the years 1980–2019, 14 million infants missed pentavalent first dose due to lack of access to routine vaccines globally [2, 6]. One in five children in Africa do not access lifesaving vaccines [7]. In 2017, facilities in South Africa experienced stock-outs of routine vaccine for over 2 weeks [8]. In Hoima Uganda, facilities experienced inadequate or lack of vaccines to meet customer demand [9]. In Kenya, healthcare facilities experience vaccine shortages [10]. Healthcare facilities in Tana River County experienced routine vaccine stock-out in the year 2020 [10]. Routine vaccine stock-out has consequences such as high dropout rates, defaulters and low immunization coverage [4, 11, 12]. Lack of vaccines increases childhood morbidity and mortality [9]. An estimated 3 million children die yearly in Africa and 24% are due to VPD [13]. In Kenya a total of 22,013 measles cases were reported between the years 2018–2021 and among them, 960 were from Tana River County. The availability of routine vaccines in healthcare facilities is crucial in ensuring high immunization coverage and preventing the burden of VPD. Tana River County face challenges related to routine vaccine stock-outs, leading to low immunization coverage rates, increased childhood morbidity among children under one year and the potential for disease outbreak. No study has been conducted to determine the factors contributing to routine vaccine stock. Identifying the factors influencing vaccine availability in Tana River County is essential for developing targeted interventions and improving the overall immunization system to protect the health of children under 1 year of age.

## Methods

### Study area and period

The data were collected between October 24th to November 25th, 2022, from immunizing healthcare facilities in Tana River County. The county is located in the coastal region of Kenya and covers an area of 38, 862 km^2^. It borders five counties and administratively subdivided into five sub-counties. It has a population of about 352,891 with 12,704 children under 1 year of age. The county has 61 healthcare facilities offering immunization services.

### Study population and sampling

The target population comprised 75 healthcare facilities and 61 of them offering immunization services were studied. Census method was used to obtain the sample size where all the immunizing healthcare facilities were included. One respondent from each immunizing healthcare facility handling vaccine inventory was selected. Simple random sampling was used to select the respondents in health facilities having more than one person offering immunization services. In facilities with only one staff, that person filled the questionnaire. All public, private and faith-based healthcare facilities providing immunization services were included.

### Data collection tools

A structured questionnaire with four sections containing various variables was used. They included the biodata, status of vaccine stock, routine vaccine stock management practices and the status of the cold chain equipment.

### Data collection procedures

A data collection plan was developed. Two research assistants were trained on the content and how to administer the structured questionnaire. This was followed by pre-testing of the questionnaire among ten healthcare workers providing immunization services. Adjustment of the questionnaire was done following the pre-testing results. Sixty-five copies of the adjusted questionnaire were printed. A final data collection schedule was prepared and shared with the research assistants. The selected facilities were visited by the researcher and his assistants. The respondents were approached at their place of work and the study explained to them. Those who were selected and consented to participate were given the questionnaires to fill and assisted where necessary. The filled questionnaires were collected and kept in a secured cabinet to ensure confidentiality awaiting further processing.

### Data management, analysis and quality assurance

The administered questionnaires were checked for any incompleteness, inconsistencies, missing values, outliers and errors and appropriate action taken. Coding of data was done and entered in an excel sheet. The sheet was exported to STATA version 14 for analyses. Descriptive analysis was done using measures of central tendency and proportions. Inferential analysis was carried out using Pearson Chi-square and Fisher’s exact as well as logistic regression at 0.05 level of significance.

## Results

### Socio-demographic characteristics

Out of the 61 targeted samples, 59 responded representing 96.7% response rate. Of the 59 participants interviewed 62.71% were male and 64.4% were below 40 years of age. Nurses comprised the largest proportion of the participants at 96.61% and majority were diploma holders at 77.97%. The results further revealed that majority (64.4%) of the respondents had worked for more than two years in the immunization department with only 49.15% having been trained on operational/mid-level management course on immunization.

The relationship between socio-demographic characteristics and availability of vaccines was assessed at 0.05 level of significance. This was done using Pearson Chi-square or Fisher’s exact. Statistically significant relationship was observed between availability of vaccines and working experience (*p* = 0.001), training (*p* = 0.024) as well as age of the participants (*p* = 0.016).

### Factors affecting availability of routine vaccines

Majority (62.71%) of the facilities experienced routine vaccine shortage. Vaccine stock-out at the district deport was the main contributing factor to routine vaccine shortage at 27.12%. Other factors were under estimation of vaccine requirements (15, 25%), delay in placing order (13.56%) and faulty refrigerator (6.78%). The vaccines that were assessed had varied levels of stock to complete the supply period**.** The one that had the largest quantity to complete the supply period was Measles–Rubella (71.19%) followed by BCG (69.49%), Penta Valent (67.8%) and lastly oral polio vaccine (57.63%). Statistically significant relationship was observed in delay in placing order (*p* = 0.02) and underestimating vaccine requirement (*p* = 0.02).

### Types of stock management practices

It was observed that all health facilities had target population for immunization displayed but only 55.93% had area catchment map with target population (Table 1). Target population method was widely used for forecasting yearly vaccine requirements at 35.59%. In addition, 23.73% of the facilities had correctly filled vaccine forecasting sheet and 89.83% ordered vaccines when the stock reached minimum levels. Majority (81.36%) of the respondents used one month as the supply period and 76.27% had correctly filled vaccine ledger book.

**Table 1:** Types of stock management practices

Management practices	Number of facilities	Percentage
*Availability of information on target population*		
Area catchment map with population targets displayed	33	55.93
Facility monthly population target available	59	100
*Methods used to forecast vaccine requirements*		
Target population	21	35.59
Consumption method	18	30.51
Both target population and consumption methods	11	18.64
Estimation	9	15.25
Availability of accurately filled vaccine forecasting sheet	14	23.73
*Factors that trigger placing a vaccine order*		
Vaccine stock reaching minimum levels	53	89.83
Vaccine stock reaching maximum levels	2	3.39
Vaccines stock depletion	2	3.39
Opportunity arising regardless of current stock levels	2	3.39
*Supply interval for vaccines in the facilities in months*		
One	48	81.36
Two	5	8.47
Three	6	10.17
*Staff knowledge on how to calculate vaccine requirement*		
Use of consumption method	35	59.32
Use of target population method	28	47.46
Ability to determine the quantity to order at any given time	38	64.41
*Status of the last order*		
Exceeded the maximum stock for the order period	18	30.51
Correct quantity for the order period	25	42.37
Under stocking for the order period	16	27.12
*Stock management practices*		
Vaccine order form/ s11 used	29	49.15
Correctly and completely filled vaccine ledger book	40	67.80
Vaccine arranged according to WHO recommendation	48	81.36
Monthly vaccine physical count conducted	45	76.27

The relationship between vaccine stock management practices and availability of vaccines was assessed at 0.05 level of significance. Statistically significant associations between availability of vaccines were observed with display of area catchment map (*p* = 0.045), knowledge on how to calculate vaccine requirement using target population (*p* = 0.003) as well as status of last order (*p* = 0.004).

### Monitoring of vaccine use and immunization operating standards and services

The monitoring of vaccine use and presence of standard operating procedure booklet were investigated and the results are shown in Table 2. Most of the respondents (93.22%) prepared monthly summary report and 83.05% used immunization monitor chart. Majority (86.44%) of the participants had the correct knowledge on the supply period required for a facility.

**Table 2:** Monitoring of vaccine use and immunization operating standards procedures

Variable	Number of participants	%
*Methods of monitoring vaccine use*		
Calculated vaccine wastage rates at facility	39	66.10
Monthly summary report of vaccine stock	55	93.22
Use of immunization monitoring chart	49	83.05
Immunization service monthly review meeting	34	5.63

Operating standard or service	Frequency	Percent
Availability of immunization SOP booklet	17	28.81
Time taken to retrieve immunization SOP booklet in minutes		
< 5	12	20.34
5–10	5	8.47
Number of months of stock required in the facility		
< 1	51	86.44
> 1–2	2	3.39
> 2–3	6	10.17
Number of days immunization services are offered in a week		
One	5	8.47
Two	11	18.64
Five	43	72.88
Reasons of immunization services not being provided daily		
No refrigerator	3	5.08
Faulty refrigerator	3	5.08
Workload	3	5.08
To avoid wastage of vaccines	6	10.17

Variable	Frequency	Percentage
Availability of pre-qualified WHO refrigerator	56	94.91
Years in service of the refrigerator		
< 5	15	25.42
5–10	21	35.6
> 10	20	33.90
Unknown	3	5.08
Availability of adequate storage space	54	91.53
Functioning cold chain equipment	51	86.44
Number of weeks refrigerators were malfunctioning		
> 3	5	8.47
Unknown	54	91.53
Frequency of cold chain breakdown for the last 12 months		
Once	8	13.56
Twice	14	23.73
At least three times	7	11.86
None	30	50.85
Availability of adequate biomedical engineers	1	1.69
Routine cold chain equipment maintenance	12	20.34
How fast is a break down fridge repaired in weeks		
< 1	2	3.39
1–< 2	5	8.47
2–< 3	15	25.42
> 3	37	62.71

The relationship between monitoring vaccine use, immunization operating standards procedures and availability of vaccines was assessed at 0.05 level of significance. Statistically significant relationship was observed between availability of vaccines and monthly immunization service review meeting (*p* = 0.006).

### Cold chain equipment

The result showed 25.42% of the equipment was installed less than 5 years ago while 35.60% was in use between 5–10 years (Table 3). The findings revealed that 86.44% of the facilities had functioning cold chain equipment. However, 11.86% experienced breakdown more than three times in one year. It further revealed that biomedical engineers were inadequate and only 20.34% of the facilities had routine cold chain equipment maintenance.

**Table 3:** Status of cold chain equipment and maintenance

Variable	Frequency	Percentage
Availability of pre-qualified WHO refrigerator	56	94.91
Years in service of the refrigerator		
< 5	15	25.42
5–10	21	35.6
> 10	20	33.90
Unknown	3	5.08
Availability of adequate storage space	54	91.53
Functioning cold chain equipment	51	86.44
Number of weeks refrigerators were malfunctioning		
> 3	5	8.47
Unknown	54	91.53
Frequency of cold chain breakdown for the last 12 months		
Once	8	13.56
Twice	14	23.73
At least three times	7	11.86
None	30	50.85
Availability of adequate biomedical engineers	1	1.69
Routine cold chain equipment maintenance	12	20.34
How fast is a break down fridge repaired in weeks		
< 1	2	3.39
1–< 2	5	8.47
2–< 3	15	25.42
> 3	37	62.71

These variables showed no statistically significant relationship with availability of vaccines

### Predictors of vaccine availability

Logistic regression was used to determine the predictors of the availability of vaccines (Table 4). The test was assessed at 0.05 level of confidence. The independent variables were chosen among those exhibiting *p*-values less than 0.25 following Pearson chi square and Fisher’s exact tests. A bivariate analysis revealed a statistically significant association between availability of vaccines and work experience, training on immunization services, catchment area map with target population displayed in the facility, and use of target population method in vaccine forecasting. The independent predictor on multivariate analysis was work experience (AOR = 2.65, 95%CI, 1.13, 6.25, *p* = 0.025). Facilities where the participants had more than two years of experience in the immunization department had 2.65 odds of having all the childhood vaccines available compared to those where the experience was less than two years.

**Table 4:** Predictors of vaccine availability

Independent variables	Bivariate analysis COR,95%CI	*p*-value	Multivariate analysis AOR, 95%CI	*p*-value
Work experience – ≤ 2 years vs > 2 years	3.48 (1.71, 7.09)	0.001*	2.65 (1.13, 6.25)	0.025*
Age – ≤ 40 years vs > 40 years	1.77 (1.00, 3.14)	0.052	1.05(0.47,2.37)	0.899
Training on operational level training or mid-level management – Yes vs no	3.52 (1.15, 10.75)	0.027*	1.60(0.31,8.29)	0.577
Catchment area map with target population displayed in the facility – Yes vs no	3.13 (1.00, 9.8)	0.049*	2.67(0.57,12.54)	0.213
Use of target population method for vaccine forecasting – Yes vs no	5.56 (1.74,17.79)	0.004*	1.33 (0.20,8.69)	0.770
Ability to calculate quantity of vaccines required at a point in time – Yes vs no	2.59 (0.79,8.51)	0.117	1.62(0.25,10.52)	0.614
Vaccine order status *– *Excess vs low	0.500 (0.24, 1.05)	0.068	0.53 (0.19, 1.5	0.232
Vaccine ledger book correctly filled – Yes vs no	3.07(0.86,10.89)	0.083	1.72 (0.29, 10.15)	0.548
Calculation of vaccine wastage rates done – Yes vs no	3.43(0.97,12.13)	0.056	2.24 (0.42,12.02)	0.347
Strategically displayed vaccine consumption chart – Yes vs no	1.69 (0.51, 5.56)	0.387	0.85 (0.17, 4.36)	0.847

## Discussion

The purpose of the study was to find out the factors that influence the availability of childhood routine vaccines in healthcare facilities at Tana River County.

### Status of vaccine stock

Over a half of the facilities experienced childhood routine vaccine stock-outs and the main reason was work experience. Facilities where the respondents had more than two years work experience were more likely to have the vaccines. Experience enhances competence and effectiveness. The other factors that were statistically significant to vaccine availability included training of healthcare workers, use of target population during forecasting and presence of the catchment area map. Underestimating routine vaccine requirement, delay in placing order and faulty refrigerator contributed to stock-out thereby potentially increasing prevalence of childhood VPD. These results provide an insight of existing gaps in vaccine supply chain, poor practices on vaccine stock management and weak cold chain system. The findings partially concur with studies done in Kenya and South Africa [10, 14]. The selected vaccines for assessment had varied levels of stock that could last the supply period. This is a further confirmation of possible issues on vaccine supply and vaccine stock management issues. Vaccine stock-out and rationing at the sub-county vaccine depot were major causes of the vaccine shortages [10].

### Vaccine stock management practices

In practice, the results revealed that half of the facilities display area catchment map and used target population method for yearly vaccine forecasting. However, the findings showed that only 23.73% had correctly filled vaccine forecasting sheet. Further results revealed that majority ordered vaccines when stock levels reached its minimum and practiced one month as their supply period. The inability to forecast vaccine requirement at a point in time, delay in placing orders and underestimating of vaccine requirements suggest a knowledge gap in vaccine stock management. These findings concur with previous study which shows that inaccurate knowledge on forecasting contributed to vaccine stock-out at the facilities [10]. These observations affirm that poor vaccine stock management practices affect vaccine visibility. Training increases the level of knowledge and skills which are essential in forecasting the quantity required and ability to calculate the quantity of vaccines at a point in time. The results further revealed that slightly above half of the respondents had correctly filled vaccine ledger book. This provides an insight of inadequate vaccine accountability. Despite this, majority of the participants conducted monthly physical count, prepared monthly summary, monitored immunization coverage and conducted monthly review meeting. The monitoring of vaccine use is essential in ensuring vaccine visibility.

### Status of cold chain

Almost all the facilities had a refrigerator for storing the vaccines which concurs to a similar study carried out in Ethiopia [15]. Majority of the cold chain equipment were installed and in use for more than 5 years. A good percentage of the equipment were installed and in use for more than 10 years. This exposes them to frequent breakdown. The breakdown affects storage as vaccines are biological products requiring special storage conditions. It was also observed that biomedical engineers were inadequate. This affected the routine maintenance of the cold chain equipment further exposing them to the risk of breaking down. The number of immunizing health facilities, the vastness of the County in relation to the availability of biomedical engineers could have contributed to ineffective routine maintenance.

The logistic regression results revealed that working experience had statistically significant relationship on vaccine availability. The facilities whose staff had worked more than two years were 6.25 more likely to have the vaccines than those facilities whose staffs had worked less than two years. Display of area catchment map with target population is important. It serves as a reminder on the population to be covered. Staff develop mastery of movement trends in and out of the catchment area which is key information in vaccine forecasting and quantification. Working for many years provides the necessary exposure, building knowledge and skills over time. The knowledge includes the ability to calculate the quantity of vaccine requirement at a point in time, accurate forecasting, timely ordering and accurate vaccine documentation in the ledger book. Work experience in the field of vaccine stock management enhances vaccine visibility.

The availability of vaccines was mainly dependent on both human and institutional factors. Optimizing stock levels in health facilities at Tana River County will require adequate training of employees on quantification and maintenance of the cold chain equipment. Appropriate inventory management at the district deport is essential to prevent stock-outs.

### Limitation of the study

The study was conducted in a rural set-up (Tana River), and therefore the results cannot be generalized in an urban set-up as well as other rural regions.

## Conclusion

Healthcare facilities experienced routine vaccine stock-out which was attributed to poor vaccine stock management practices and weak cold chain system. Work experience contributed significantly to availability of vaccines in health facilities.

## Data Availability

The data used to produce the current manuscript are available upon a reasonable request to the corresponding author.

## Abbreviations

AOR: Adjusted odds ratio
BCG: Bacillus Calmette Guerin
CI: Confidence interval
COR: Crude odds ratio
MR: Measles–Rubella
OPV: Oral Polio Vaccine
VPD: Vaccine preventable diseases
